# Supplementary material for: Body fat percentage is a key factor in elevated plasma levels of caffeine and its metabolite in women
Source: PeerJ. 2025 Jun 9;13:e19480. doi: 10.7717/peerj.19480 (PMC12161129; doi:10.7717/peerj.19480)
Supplement: Supplemental Information 1 [file peerj-13-19480-s001.docx]

**Trial Protocol**

**Obesity as a key factor in elevated plasma levels of caffeine and its metabolite in women**

**1. Study Objective**

- To investigate the relationship between body fat percentage and plasma concentrations of caffeine and its primary metabolites, specifically in women, to determine if caffeine dosing should be based on body fat percentage rather than body weight.

**2. Study Design**

- Type: Randomized, controlled study with an observational component.

- Duration: Single visit for measurement and sampling.

- Participants: Thirty-eight women aged 22-31 years, categorized into two groups based on body fat percentage: non-obese (≤30%) and obese (>30%).

**3. Eligibility Criteria**

- Inclusion Criteria: Women aged 18-31, non-smokers, caffeine consumers with no hypersensitivity, no medical conditions or medications affecting caffeine metabolism, and informed consent.

- Exclusion Criteria: Smoking, caffeine hypersensitivity, medical contraindications, medications affecting caffeine metabolism, pregnancy, or breastfeeding.

**4. Randomization and Blinding**

- Participants were divided based on pre-defined body fat percentage thresholds, with no randomization for intervention (caffeine dose was fixed). Blinding was not feasible due to the single-dose caffeine administration based on body weight.

**5. Intervention**

- Dose: 6 mg of caffeine per kilogram of body weight, administered via cellulose capsules.

- Timing: Blood samples were taken 60 minutes post-ingestion to capture peak caffeine and metabolite concentrations.

**6. Outcomes**

- Primary Outcome: Plasma concentration levels of caffeine and paraxanthine, measured via high-pressure liquid chromatography (HPLC).

- Secondary Outcome: Any self-reported side effects and the relationship between caffeine levels and body fat percentage.

**7. Data Collection and Analysis**

- Plasma samples were analyzed for caffeine and its metabolites (paraxanthine, theobromine, and theophylline) through LC-MS/MS.

- Statistical Analysis: Mann-Whitney U test was used due to non-parametric data distributions. The median and interquartile range were calculated.

**8. Ethics and Consent**

- Approval: Bioethics Committee of the Poznan University of Medical Sciences.

- Consent: Informed consent obtained from all participants, with assurance of the right to withdraw at any time without consequence.

**9. Safety and Adverse Events**

- Monitoring for any acute adverse reactions to caffeine and adherence to exclusion criteria to mitigate risks.
